# Preparation of starch-based functional food nano-microcapsule delivery system and its controlled release characteristics

**DOI:** 10.3389/fnut.2022.982370

**Published:** 2022-08-15

**Authors:** Shuangqi Tian, Xing'ao Xue, Xinwei Wang, Zhicheng Chen

**Affiliations:** College of Food Science and Technology, Henan University of Technology, Zhengzhou, China

**Keywords:** functional food, starch, nano-microcapsule, release characteristic, delivery system

## Abstract

Most of the functional substances in food are absorbed in the small intestine, but before entering the small intestine, the strong acid and enzymes in the stomach limit the amount that can reach the small intestine. Therefore, in this paper, to develop a delivery system for functional food ingredients, maintain the biological activity of the ingredients, and deliver them to the target digestive organs, preparation of starch-based functional food nano-microcapsule delivery system and its controlled release characteristics were reviewed. Embedding unstable food active ingredients in starch-based nano-microcapsules can give the core material excellent stability and certain functional effects. Starch-based wall materials refer to a type of natural polymer material that uses starch or its derivatives to coat fat-soluble components with its hydrophobic cavities. The preparation methods of starch-based wall materials mainly include spray drying, extrusion, freeze drying, ultra-high pressure, coagulation, fluidized bed coating, molecular inclusion, chemical, and enzymic methods. The controlled release of functional food can be achieved by preparing starch-based nano-microcapsules to encapsulate the active agents. It has been reported that that compared with traditional embedding agents such as gelatin, acacia gum, and xanthan gum, starch-based functional food nano-microcapsule delivery system had many good properties, including improving antioxidant capacity, bioavailability, probiotics, and concealing bad flavors. From this review, we can learn which method should be chosen to prepare starch-based functional food nano-microcapsule delivery system and understand the mechanism of controlled release.

## Introduction

More and more attention has been paid to the design and application of foods containing biologically active substances with natural functional ingredients (1, 2). Different drugs, dietary supplements, and functional food ingredients, as part of the daily diet, play an increasingly important role in the prevention of common chronic diseases, such as diabetes and hypertension (3–5). With the development of the food industry and the enhancement of people's health awareness, the development of foods containing functional ingredients has become one of the hot spots in the food industry (6, 7). Although natural food active ingredients have many benefits to human health, they are easily affected by environmental factors, such as oxygen, moisture, temperature, pH, etc., which easily lead to their degradation or inactivation, and limit their applications in the food industry (8, 9).

**Figure 1:**
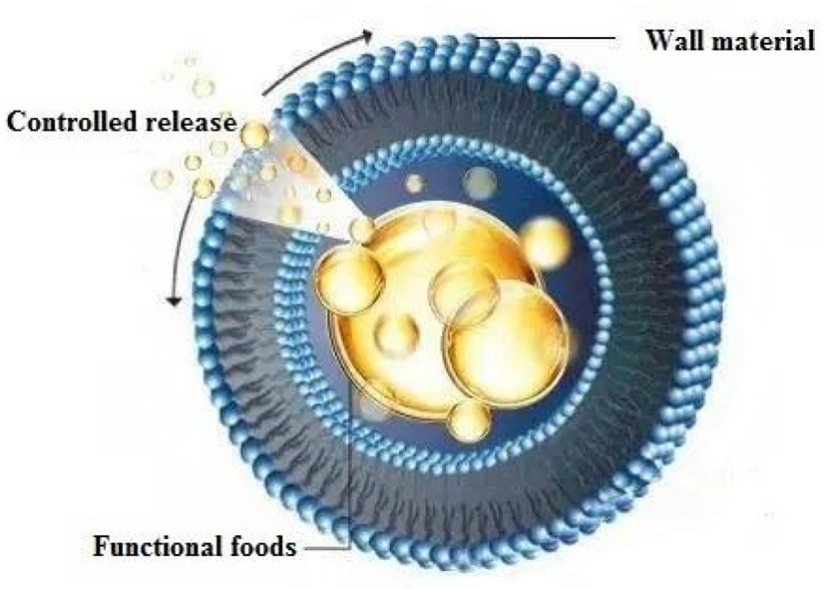
Schematic diagram of nano-microcapsule controlled release process.

Nano-microencapsulation technology was first used in the pharmaceutical industry, and then gradually expanded to the food industry, becoming a commonly used technology in today's food production (10–12). As shown in Figure 1, nano-microencapsulation technology is to provide protection for the functional foods and enhance their solubility, dispersion characteristics and bioavailability by wrapping biologically active substances such as phenols, flavonoids, anthocyanins, vitamins, and fatty acids in the wall material of the nano-microcapsules (13–15).

Starch is a renewable biopolymer widely distributed in nature and has a wide range of industrial application prospects (16–18). Starch has been used as packaging material to protect compounds such as herbicides, proteins, microorganisms, probiotics, etc. (19–23). The wide application of starch in microencapsulation is based on its high availability, low cost and multiple functions (water retention, high or low viscosity, etc.) (24, 25). Different starch-based microcapsule embedding wall materials, including cyclodextrin, amylose, OSA-modified starch, etc., can be applied to the embedding of various food active ingredients. A new type of starch-based embedded wall material can be developed through the regulation of modification sites, and its application range can be expanded (26–36).

Starch can be used as a carrier to protect the core material, encapsulate and target the guest molecules, controllable release, or mask unpleasant flavors such as acid, astringency, and bitterness (37–39). However, natural starch is low in utilization due to its poor solubility and weak water holding capacity, which limits its application in other fields. Currently, many studies have confirmed that natural starch should be modified reasonably, by controlling the aggregate structure and chain structure of starch, increasing the content of amylose, giving starch lipophilic groups, and changing its solubility, water absorption and other properties, so that it becomes a good wall material for nano-microcapsules (39, 40). Starch-based nano-microcapsule delivery systems allow for the protection and delivery of food ingredients that have health benefits but are unstable during processing, storage and in the upper gastrointestinal tract (41, 42). This article will introduce the types of starch-based microcapsule wall materials, the formation mechanism, the technical route of preparation and the method of structural characterization, focusing on its application in the embedding of functional foods and other nutrients, including the embedding process, structural characterization and release evaluation.

## Preparation of starch-based functional food nanomicrocapsule delivery system

According to its own characteristics, starch is usually modified by physical, chemical or enzymatic methods to improve or change its inherent physiochemical properties, so as to produce composite nano-microcapsules with higher yields while retaining the functional and physicochemical properties of natural starch.

### Physical methods

#### Microwave

Compared with traditional methods, microwave technology has the advantages of saving energy and time, reducing the use of organic solvents, simplifying operating procedures, increasing the heating rate, and significantly reducing the environmental hazards. Microwave technology is not only widely used in starch food processing, such as food heating, enzyme inactivation, baking, thawing, puffing, sterilization, etc., but also the application of microwave technology for the modified starch has gradually become a hot spot, such as in microwave ovens carrying out starch esterification, hydrolysis, oxidation and affinity reaction, the speed is several times, tens of times or even hundreds of times higher than traditional methods (43–45). Short microwave heating of starch granular from potato, waxy corn and tapioca with such lipids as *cis,cis*-9,12-octadecadienoic acid (linoleic acid), *cis*-9-octadecenoic acid (oleic acid), octadecanoic acid (stearic acid), ethyl cis-9-octadecenoate, ethyl *cis,cis*-9,12-octadecadienoate and methyl octadecanoate provided nano-microcapsules in which encapsulated guest molecules did not interact with starch nano-microcapsules. On the formation of nano-microcapsules, the lipid guest molecules did not react to starches. The encapsulation yield varied between almost 11–94% (46).

#### Spray drying

Spray drying is a widely used microencapsulation technology, which has the characteristics of simple operation and low cost. The principle of spray drying is to pass liquid raw materials through an atomizer to form many tiny droplets. The droplets directly contact the dry hot air, and quickly evaporate to form dry tiny particles, which are collected in a container (47, 48). 20 g of starch and almond oil was dispersed for 10 min using a disperser, and finally dried it with a spray dryer. Continuous stirring during the drying process ensured that the material was uniform and collects the solids to obtain starch-almond oil nano-microcapsules. The results of their morphology and physicochemical stability showed that almond oil was mainly located in the internal cavity of spherical aggregates, and spray drying was in the process of microencapsulation. The peroxide value of almond oil was increased, and the chemical stability of almond oil was improved. Therefore, microencapsulation inhibited the oxidation reaction of almond oil and enhanced its antioxidant properties (29). *n*-Octenylsuccinate-derivatised starch was shown to be an interesting material for the encapsulation of passion fruit juice, and spray drying proved itself as an inexpensive alternative to freeze-drying, capable of retaining vitamin C during a long time of storage, and easy to be diluted in order to reconstitute the passion fruit juice for human consumption (49).

#### High pressure homogenization

To produce strong shear, impact and cavitation to destroy the starch granules, the high-pressure liquid pass through the small gaps of the homogenizer valve, so that the amylose is dissolved and compounded with the guest molecules to form a complex. Lotus seed starch and tea polyphenols were dispersed in distilled water, repeated treatments through a high-pressure homogenizer (60–180 MPa). The eluted excess tea polyphenols were finally freeze-dried to obtain starch-tea polyphenol nano-microcapsules. The results showed that the nano-microcapsules exhibited a C-type crystal structure and a “net-like” surface structure at a pressure lower than 150 MPa (50). Lysolecithin and peanut starch were mixed under high pressure homogenization conditions to obtain similar starch nano-microcapsules. The starch-lysolecithin formed a V-shaped crystal structure after high pressure homogenization. The structure of the starch increased the water holding capacity and enthalpy value of the starch, and decreased the viscosity. The homogenization treatment significantly increased the complexation between peanut starch and lysophosphatidylcholine, which reduced the syneresis rate of the nano-microcapsules. The results showed that the starch nano-microcapsules prepared by the homogenization method could be used in frozen foods and dessert foods with the smooth texture (51).

#### Ultrasonic waves

The main effects of ultrasonic waves in liquids are mechanical shearing and cavitation. The cavitation of ultrasonic waves can produce instantaneous high temperature and produce bubbles. The collapse of bubbles produces a strong mechanical effect, which can cause the destruction of some structures of starch (52, 53). Among many methods, the ultrasonic treatment method is a convenient and effective method to synthesize starch nano-microcapsules. The ultrasonic treatment method has simple operating conditions, relatively mild reaction conditions, simple equipment, short reaction time, and no need for additional high temperature and high pressure, but the resulting product has excellent targeted delivery characteristics and stimulus response control characteristics (54, 55). Zhu et al. prepared jackfruit seed starch-vanilla essential oil nano-microcapsules using ultrasound. The vanilla essential oil was dissolved in a starch supersaturated solution and lyophilized after ultrasonic treatment. The yield and loading rate of the nano-microcapsules were 84.82 and 79.33%, respectively. The capsule has good oxidation resistance, storage stability, and slow release potential (56). The ultrasonic treatment could decrease the particles size of chitosan-SiO_2_ particles. The energy disperse spectroscopy of samples with ultrasonic treatment for 0 min and 10 min indicated that the SiO_2_ was present in prepared chitosan-SiO_2_ particles. When the ultrasonic time reached 10 min, the chitosan-SiO_2_ nanoparticles formed and the particle size was 506.7 nm. Zeta potential of the chitosan-SiO_2_ nanoparticles was >30 mV, showing the suspension was a stable dispersed liquid. The results showed that chitosan-SiO_2_ nanoparticles could be used as a reinforcement to prepare thermoplastic starch films and promoted the application of chitosan nanoparticles in nanocomposite films (57).

#### Ultra-high pressure

Ultra-high pressure technology is a kind of non-thermal processing technology. Water was used as a medium in a normal temperature or low temperature environment to process sample materials by applying a pressure of more than 100 MPa, compressing the macromolecular substances in the food, and changing interaction forces (including hydrogen bonds, ionic bonds and hydrophobic interactions, etc.) to achieve the purpose of modification (58). A novel type of casein-porous starch microgel was prepared under ultra-high pressure, by using porous starch with the honeycomb three-dimensional network porous structure. Molecular interaction force analysis and thermodynamic analysis showed that electrostatic interaction played a major role in the formation of microgels. Compared with casein-glutinous rice starch microgels, the encapsulation efficiency and loading capacity of phycocyanin in casein-porous starch microgels were increased by 77.27 and 135.10%, respectively (59). The ultra-high pressure method was used to prepare lotus seed starch-fatty acid nano-microcapsules. The lotus seed starch and fatty acid were dispersed in a 10% ethanol solution, and a hydrostatic pressure of 600 MPa was applied at 25°C. The particles was collected to obtain lotus seed starch-fatty acid nano-microcapsules. The results showed that the nano-microcapsules under ultra-high pressure presented a typical V6 type polymorph, and fatty acids were present in the amylose helix and partly in the amorphous region of the starch (60).

#### High-speed shearing

The preparation principle of the high-speed shearing method is that the starch particles are pulverized through the process of grinding, shearing and collision, etc., and the factors that affect the pulverization level include the concentration of starch milk, the rotation speed, and the grinding beads.

Starch-based nano-microcapsule delivery system prepared by the high-speed shearing method have the characteristics of irregular shape and small crystallinity, which makes the dilution amount and the dissolution rate improved, and the gelatinization process can be easily realized at room temperature. The high-speed shearing method can replace the role of chemical reagents, and has the advantages of high efficiency and easy operation, but it also has its shortcomings. It consumes more energy during the operation, which is also the reason for its less industrial application. Potato starch nanocapsules with an average particle size of 120 nm were prepared by a high-energy ball mill using ZrO_2_ as a medium (61).

#### Freeze drying

Freeze drying method is a process in which materials are frozen under vacuum conditions, and then a very small amount of heat is applied to directly sublime the moisture in the materials. The freeze-drying method prepares nano-microcapsules by mixing core material and wall material solutions, and obtaining a stable and uniform aqueous solution through the operations such as stirring and homogenization (28, 32, 62). The aqueous solution is freeze-dried to obtain dry and irregular nano-microcapsules. Freeze drying method is suitable for water-soluble core materials and wall materials, especially for core materials that are extremely unstable to heat. Freeze drying method can also assist other nano-microcapsule preparation methods to remove moisture from materials (32). Maltodextrin was used as the wall material, and freeze-drying proanthocyanidin nano-microcapsules could be stored for 2 months under the conditions of 50°C and A_w_ = 0.5 (63).

### Chemical methods

#### Esterified starch

Esterified starch uses an esterifying agent such as octenyl succinic anhydride (OSA) to esterify partial functional groups of starch in an alkaline environment to prepare OSA modified starch. Compared with natural starch, OSA modified starch has good hydrophilicity, emulsification and gel characteristics. It is a new, safe, low-cost emulsifier and thickener. Due to the high concentration and low viscosity of OSA starch aqueous solution, many studies have applied OSA starch to the embedding of hydrophobic food ingredients in recent years, and have achieved good results (64–66). As shown in Table 1, different countries have a regulatory limit on the amount of sodium starch octenyl succinate.

**Table 1:** Regulatory limits of sodium octenyl succinate starch %.

	**Japan**	**USA**	**EU**	**JECFA**
OSA limits	—	≤ 3.0	—	—
Occienyl succinate group	≤ 3.0	—	≤ 3.0	≤ 3.0
Residual ocenyl succinate	≤ 0.8	—	≤ 0.3	≤ 0.3

Gum arabic and OSA modified sorghum starch matrix was used to embed nutmeg oleoresin. The starch was acetylated and mixed with nutmeg oleoresin into a solution, and the nutmeg oleoresin-modified starch nano-microcapsules were prepared using a spray dryer. It was found that compared with the original starch, the modified starch nano-microcapsules had excellent biological activity and functional properties. The results showed that the nano-microcapsules had excellent antioxidant activity and high retention rate of phenols and flavonoids after 60 d of storage, and exhibited resistance to *Escherichia coli* and *Bacillus cereus* (67). β-carotene (BC) and eugenol (EU) co-encapsulated flax seed oil (FSO) emulsions stabilized by octenyl succinic anhydride modified starches (OSA-MS) were dried to powders after the emulsification process. Nano-microcapsules showed good dissolution behavior, high (≈90%) microencapsulation efficiency and semi-spherical morphology observed by scanning electron microscopy. The results indicated a positive role of EU as antioxidant and low Mw OSA-starch as wall material for the encapsulation of lipophilic bioactives that could be used for the development of functional foods and beverages (68).

#### Oxidized starch

As an important method of starch modification, oxidized starch mainly oxidizes the hydroxyl groups at carbon positions 2, 3, and 6 of starch molecules to carbonyl and carboxyl groups. The oxidation could destroy the glycosidic bonds that connect the starch molecules, and change the structure of starch molecules, resulting in a series of changes in its properties (35, 69). After starch is oxidized, its molecular weight, crystalline structure, gelatinization temperature and gelatinization absorption enthalpy undergo a series of changes, and it has the advantages of anti-aging and thermal properties (70, 71). Corn starch was oxidized with sodium hypochlorite to enhance its film-forming properties and reduce viscosity. This oxidized starch (OS) was further modified by n-octenyl succinic anhydride (OSA) to enhance hydrophobicity. This dual modified starch (OS-OSA) had the characteristics of crystallization and thermal properties. The emulsion stability, viscosity, and particle size of an OS-OSA-based emulsion containing 60% soybean oil was compared with OS and gum arabic (GA). Using OS-OSA and GA as wall materials, spray-dried nano-microcapsules containing 20% (w/v) emulsion showed similar surface morphology and particle size (69). Spray-dried gelatin/oxidized corn starch (G/OCS) nano-microcapsules were used in functional food delivery. The prepared nano-microcapsules were characterized by scanning electron microscope (SEM) images and thermogravimetric analysis (TGA). The swelling properties of G/OCS nano-microcapsules and the release properties of vitamin C were studied. The structural analysis results showed that there was miscibility and compatibility between oxidized corn starch and gelatin, and exhibited high thermal stability up to 326°C. The swelling of G/OCS nano-microcapsules increased with increasing pH and decreased with decreasing ionic strength. This was attributed to the cross-linking between gelatin and oxidized corn starch and the ionization of functional groups. The release characteristics of vitamin C showed a controlled release behavior within the first 3 h of contact with the aqueous medium (35).

#### Acid hydrolyzed starch

Acid hydrolyzed starch refers to a type of modified starch obtained by treating natural starch with mineral acid below the gelatinization temperature and changing its properties. The acid hydrolyzed starch has low viscosity and can prepare high-concentration paste (72, 73). Starch microspheres (SMs) were prepared in an aqueous two-phase system (ATPS). A series of starch samples with different molecular weights were prepared by acid hydrolysis, and the influence of starch molecular weight on the preparation of SMs was studied. SEM showed that the morphology of SMs changed with the molecular weight of starch. When the starch samples with weight-average molecular weight ≤ 1.057 × 10^5^ g/mol were used, spherical SMs with sharp contours were obtained. X-ray diffraction (XRD) results showed that the crystal structure of SMs was different from that of natural tapioca starch, and the relative crystallinity of SMs increased with the decrease of starch molecular weight. Differential scanning calorimetry (DSC) results showed that peak gelatinization temperature (Tp) and enthalpy of gelatinization (ΔH) of SMs increased with the decrease of starch molecular weight. Stability tests showed that SMs were stable in acidic environments, but unstable under α-amylase hydrolysis (74). Phosphorylated starch was prepared and applied it to the embedding of anthocyanins. It was found that derivatization resulted in a decrease in the crystallinity and viscosity of starch. The results showed that the higher the concentration of phosphorylated starch, the better the protective effect on anthocyanins (75).

#### Cross-linked starch

The solidity of the starch particles is enhanced by chemical cross-linking, and other physical properties such as gelatinization temperature, dryness stability, shear resistance, etc. There are a large number of alcohol hydroxyl groups on the surface of starch molecules, so that there are di-ester bonds or di-acid bonds between multiple starch molecules of different sizes. The structures are connected together to obtain a cross-linked starch with a spatial network structure.

Lemos et al. aimed to use sodium trimetaphosphate/sodium tripolyphosphate cross-linked potato, banana, corn, cassava, and breadfruit starches as wall materials for C-phycocyanin encapsulation, characterize them and evaluate their *in vivo* pharmacological effects in an inflammation model. The cross-linked starches were successfully obtained, characterized, and submitted to C-phycocyanin encapsulation by freeze-drying. Among the five preparations, the cross-linked potato starch presented the highest phosphorous content (0.084%), substitution degree (0.004), water uptake capacity (0.88 g g^−1^), and C-phycocyanin encapsulation efficiency (67.58%), thus was tested *in vivo* (76).

### Enzymic methods

#### Amylolytic starch

The starch hydrolytic enzymes commonly used in the preparation of nano-microcapsules include α-amylase, β-amylase, and glucosidase. Recently, many studies have used hydrolytic enzymes to partially hydrolyze natural starch, and the formed products such as porous starch had good properties for nano-microcapsule wall material. Compared with other carrier materials, porous starch had excellent adsorption performance, because abundant pores or cavities were formed from the surface to the center of the particles, and the specific surface area was increased to form a stable pore structure. Porous starch was widely used in the food industry to embed active ingredients, such as olive oil, anthocyanins, resveratrol, vitamins, probiotics, etc. (25, 77, 78). Nano-microcapsules were developed by coating chitosan on particles prepared by crushing a mixture of extruded corn starch, resveratrol and α-amylase. In the preparation process, low-temperature extrusion and alpha-amylase were used to overcome the disadvantages of low gelatinization, low solubility and poor hydration of extruded starch. Considering the biologically active function of chitosan, the nano-microcapsules also obtained the function of starch through chitosan coating. The addition of chitosan coating and α-amylase increased the release rate of resveratrol (77). Enzyme-treated corn starch was used to prepare nano-microcapsules, and the hydrolysis time was 16 h and 20 h (ETCS 16h-GA and ETCS 20h-GA) and coated with GA. Nano-microcapsules made by GA only were used as a control. The increase in hydrolysis time lead to an increase in the number and size of pores on corn starch. The total starch content decreases with the increase of hydrolysis time, while the apparent amylose content increased. The ratio of starch/ascorbic acid was 10/1 (shell/core) (25).

#### Debranching starch

Generally, the formation of debranching starch is due to debranching enzyme treatment that causes starch to change from the entangled state to the single helical state, and the guest molecules enter the helical cavity of the starch. The hydrophobic cavity can react with many hydrophobic ligands, including alcohols, drugs, fatty acids, iodine and perfumes, to form nano-microcapsules. Therefore, the use of starch debranching enzyme to treat starch can increase the content of linear polymers in starch to increase the yield of nano-microcapsules. Debranching starch is usually treated with starch debranching enzymes, such as pullulanase and isoamylase. Debranching enzymes can directly act on the α-1,6-glycosidic bond of starch which connects to the main chain. Starch branch chains are cut to release long and short linear units of glucan (79–81). The effect of different levels (0, 4, 8 and 16 h) of pullulanase enzymatic hydrolysis on the inclusion complex between high amylose corn starch (HAMS) and ascorbyl palmitate (AP) was studied. As the debranching time increased from 0 to 8 h (the encapsulation efficiency increased to 8.63%), pullulanase pretreatment promoted the formation of complexes, resulting in an increase in enthalpy change and crystallinity. Meanwhile, the retrogradation of starch was restricted (0 and 4 h). Due to short-chain interference, excessive debranching (16 h) led to the opposite trend. *In vitro* digestibility analysis suggested that starch digestion was restrained with the increase of debranching level (resistant starch content from 29.37 to 44.59%). The formation of the composite also enhanced the stability of AP to light, heat and oxidation. Furthermore, all samples exhibited a higher release rate of AP within 1 h and persistent released curve during 1–10 h in the simulated small intestine environment (81). After the corn starch was completely gelatinized, pullulanase was added for debranching for 1 to 3 h, and then collected the debranched starch with spray-dried method. The debranched starch was dissolved in distilled water, and the xanthan gum was used as an adjuvant to homogenize after heating in a boiling water bath. Tea polyphenols were mixed uniformly and prepared nano-microcapsules with spray-dried method. The results showed that the strong gel network structure of debranched starch-xanthan gum made it more dense and viscous as the wall material of nano-microcapsules, which were conducive to the slow release of xanthan gum (82).

#### Cyclodextrin

Cyclodextrin (CD) is a cyclic oligosaccharide produced by amylose under the action of cyclodextrin glucosyltransferase produced by *Bacillus*, and usually contains 6-12 D-glucopyranose units. Cyclodextrin is a slightly tapered ring with a hydrophilic outer surface and a lipophilic cavity inside. Cyclodextrin is widely used in functional food industry because of its unique chemical structure (26, 83, 84). Folic acid was encapsulated in horse chestnut starch and β-cyclodextrin by spray-dried technology, and the release behavior of the capsule was studied under simulated gastric conditions. The nano-microcapsules showed a higher transition temperature and melting temperature than free folic acid, which indicated that folic acid was thermally stable after encapsulation. The content of antioxidants and folic acid in intestinal juice was higher than that in gastric juice, ensuring a controlled release in the intestine (26). Using β-cyclodextrin and porous starch as wall materials, allicin nano-microcapsules could be efficiently prepared by spray-dried technology. This process improved their properties by increasing their solubility, enabling them to be directly dissolved in water. In addition, the stability of allicin nano-microcapsules against heat, pH, light and oxygen has also been improved, and the content of allicin has increased by about 20% to 40%. Therefore, allicin nano-microcapsules retained the required antibacterial activity after high temperature encapsulation, and the retention rate ranged from 85.5 to 92.2% (83).

## Embedding and controlled release characteristics of starch-based nano-microcapsules

**Figure 2:**
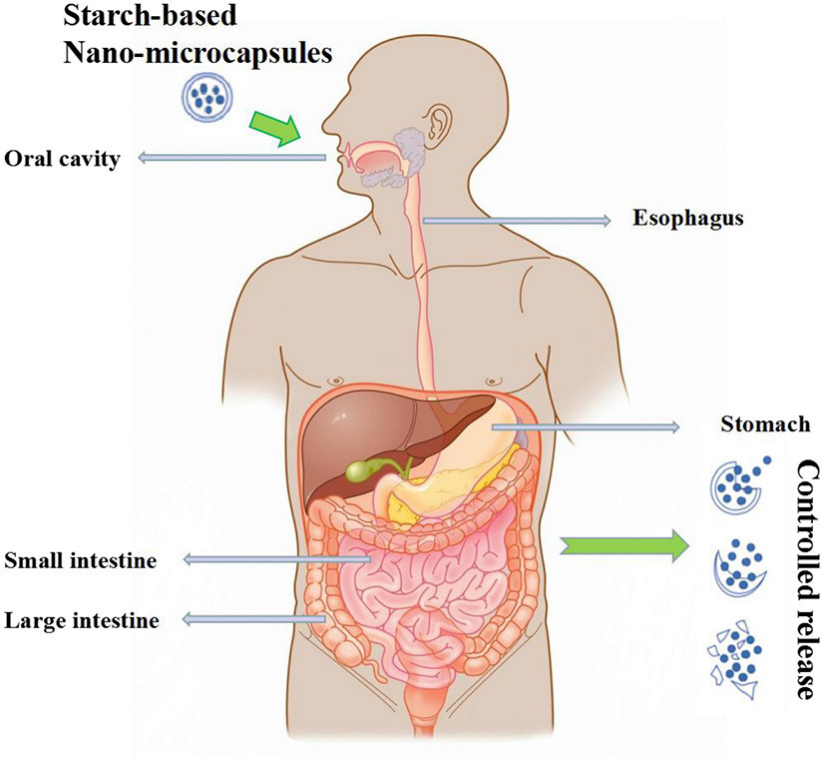
Schematic diagram of the release of starch-based nano-microcapsules in the human body.

Because the nano-microcapsules are prepared by different methods and the sources of the wall materials and core materials are different, their embedding characteristics are also different. Different sources of starches and the characteristics of starch-based nano-microcapsules prepared by different methods are shown in Table 2. The embedding characteristics of starch-based nano-microcapsules mainly include the molecular binding mode of the wall material and the core material, stability and resistance to enzymatic degradation. The molecular combination of wall material and core material is used to study and analyze its influence on the embedding rate, so as to improve the embedding efficiency of starch-based nano-microcapsules. The most important part of nano-microcapsules is that they can target the embedded biologically active substances to target organs. Therefore, the controlled release characteristics of nano-microcapsules are also an important indicator for testing the performance of nano-microcapsules. As shown in Figure 2, to deliver active substances with prebiotic effects to the target organs along the gastrointestinal tract, it must be ensured that they are not affected by gastric acid during the period. To prevent the premature release of the core material in the stomach causing it to be destroyed by gastric acid, it should be effectively absorbed in the small intestine (25).

**Table 2:** Embedding characteristics of starch-based nano-microcapsules prepared by different methods.

**Methods**	**Starch sources**	**Core materials**	**Embedding characteristics**	**Characterization methods**	**References**
Microwave	Waxy maize starch	Bovine serum albumin (BSA)	The additives PEG and BSA lowered the melting temperatures of the starch in the systems but increased the enthalpy values.	DSC	(43)
Spray drying	Taro starch	Almond oil	The almond oil was located mostly in the internal cavities of the spherical aggregates.	SEM-LV; FTIR-ATR	(29)
High pressure homogenization	Lotus seed starch	Tea polyphenols	The nano-microcapsules exhibited a C-type crystal structure and a “net-like” surface structure at a pressure lower than 150 MPa.	XRD; SEM; CLSM	(50)
Ultrasonic waves	Jackfruit seed starch	Vanilla essential oil	Storage stability and slow-releasing potential of jackfruit seed starch were more excellent than those of β-cyclodextrin and chitosan.	Optical microscope; Electronic nose	(37)
Esterified starch	Sorghum starch	Nutmeg oleoresin	The samples comprised of gum arabic and starch (native and OSA modified) in the ratio of (75:25) and (50:50) show excellent antioxidant activity and high retention of phenolic and flavonoid content after 60 days of storage.	SEM; FTIR; Antimicrobial Activity	(67)
Oxidized starch	Corn starch	Vitamin C	Vitamin C release characteristic revealed controlled release behavior in the first 3 h of contact with an aqueous medium.	SEM; TGA	(35)
Acid hydrolyzed starch	Maize starch	Purple maize anthocyanins	The highest drying yield (49.11 %) with encapsulation productivity of 87.63% resulted with 20 % of solids at 170°C.	DSC; SEM; XRD; Raman spectroscopy	(75)
Amylolytic enzyme	Corn starch	Resveratrol	The addition of chitosan coating and α-amylase increased the release rate of resveratrol, and released 86.8% resveratrol at 25°C in 6 d chasing.	SEM; XRD; FTIR	(77)
Debranching enzyme	High-amylose maize starch	Ascorbyl palmitate	The formation of complexes also enhanced the stability of AP against light, heat, and oxidation.	XRD; DSC; FTIR	(81)
Glucosyltransferase	Horse chestnut starch	Folic acid	The content of antioxidants and folic acid in intestinal juice was higher than that in gastric juice, ensuring a controlled release in the intestine.	SEM; FTIR-ATR; DSC	(26)

Simple physical treatments such as spray drying, freeze drying, high pressure homogenization, etc. trap the core material in the cavity of the starch or confine it within the starch molecules (28, 35, 58, 62). The core materials and the starch wall materials are mostly simple physical bonds. This combination can reduce the contact of the nano-microcapsules with oxygen during storage, and the contact rate with digestive enzymes, gastric acid, etc. during the digestion of the core material, thereby reducing the digestibility, and protecting the core material and providing targeted delivery. Some high-strength treatment methods such as high-pressure homogenization and high hydrostatic pressure treatment would change the crystalline structure of starch and make it form a dense structure with the core material (26, 85). For example, lotus seed starch and stearic acid formed nano-microcapsules under high-pressure homogenization conditions. High shear force caused the starch amorphous area to collapse rapidly, and the crystalline area became denser, forming a new semi-crystalline structure, showing high resistance to enzymatic hydrolysis (85). Some substances with special effects in food, such as curcumin, cinnamaldehyde, and allicin, could effectively exert their functional properties such as antibacterial or provide special flavor after microencapsulation, which could extend their validity period and make them fully function. In-depth research on the mechanism of controlled release characteristics of nano-microcapsules could not only control the production process of nano-microcapsules, but also expand their application in the food industry (86, 87). The oil-in-water microemulsion was used as a carrier to prepare nano-microcapsules. After being loaded with citral, the nano-microcapsules could achieve sustained drug release at different rates with different changes in pH (88). The results showed that 5-aminosalicylic acid, which was not embedded in resistant starch, would dissolve quickly in simulated gastric juice, and the release amount had reached 90% in 2 h. In contrast, the release rate of embedding with resistant starch was only 31%, and the sustained release effect was obvious (89).

The key factors of chemical modification affecting the embedding efficiency of starch nano-microcapsule wall materials are the content and solubility of amylose. Phosphorylation and acetylation give starch hydrophilic groups, and the esterification of hydrogen bonds limits the interaction between starch chains. Therefore, the core materials have better diffusibility in the wall materials solution system by compounding with modified starch, and the water solubility of insoluble lipids is increased. The cross-linking interactions between the carboxymethyl starch (CMS)/xanthan gum (XG) combinations and blueberry anthocyanins (ANS) were confirmed by FTIR and ^13^C solid-state NMR (90). Carboxymethyl groups had been successfully incorporated into the starch molecules. Acid hydrolysis-carboxymethyl starch (H-CMS) and XG formed a more pH-sensitive structure, which favored Vitamin E delivery from the stomach to the small intestine, especially its upper part (91). When OSA starch was compounded with XG to form a wall material, the nano-microcapsules loaded with conjugated linoleic acid (CLA) could resist the erosion of gastric acid, so that more CLA could reach the small intestine successfully (34). The intermolecular hydrogen bond formed by carboxymethyl starch and XG reduces the expansion of carboxymethyl starch-anthocyanin nano-microcapsules, making it difficult to dissociate in a low pH environment, and protecting anthocyanins from gastric acid. XG further inhibited the degradation of starch wall materials by enzymes in the digestive tract, which could delay the release of anthocyanins, and the addition of XG significantly affect the release of anthocyanins, so it could be controlled by the proportion of XG to achieve the desired release rate (92).

**Figure 3:**
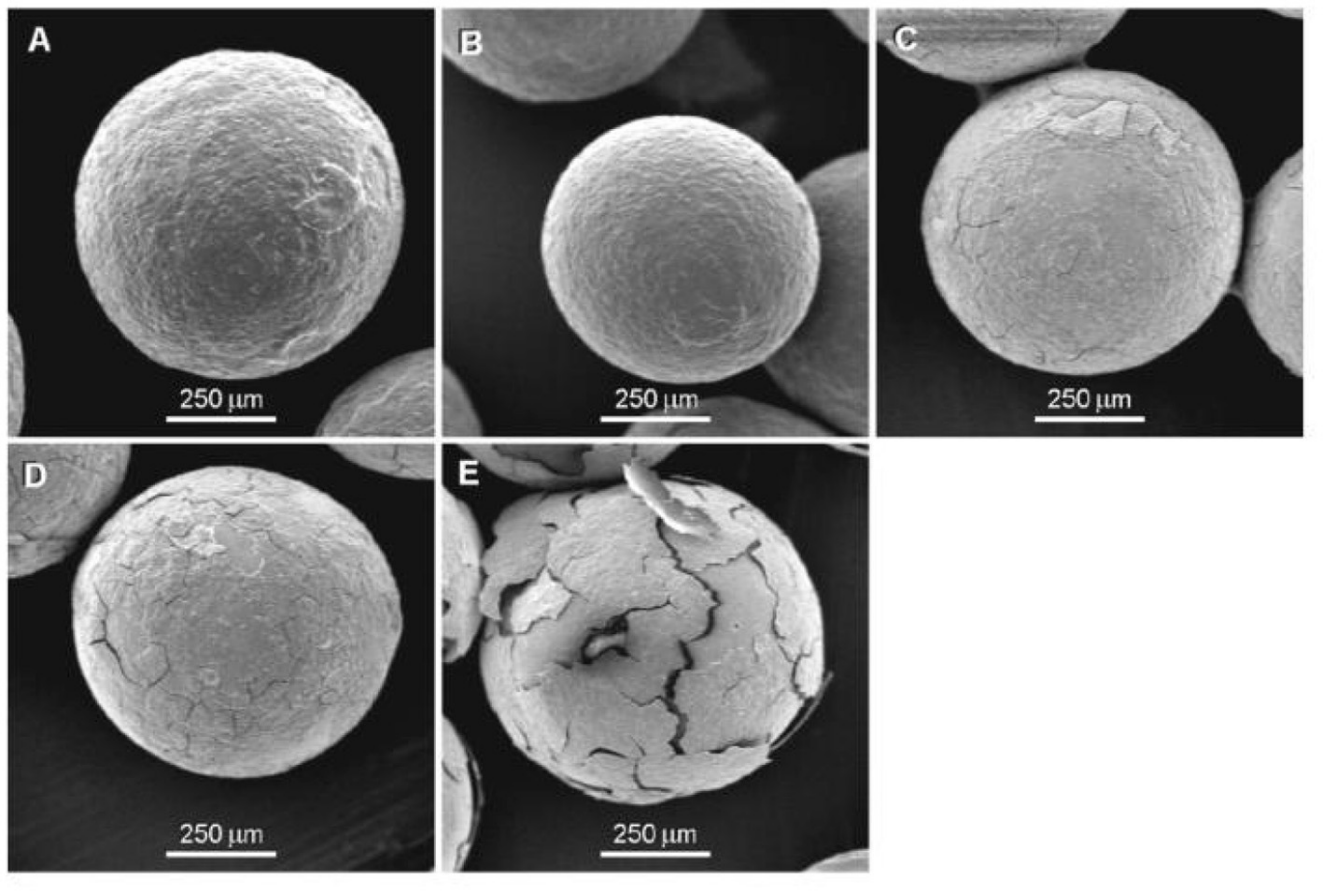
SEM images of 1.4 mgycm HACS coatings with 20% RS on glass beads. **(A)** Without treatment, **(B)** after continuous dissolution test of 2h incubation in pH = 1.6 following 3 h incubation in pH = 7.0, 37°C, 75 rpm **(C)** after 0.5 h enzymatic digestion test with 35 U/ml pancreatic a-amylase, **(D)** after 1 h enzymatic digestion test with 35 U/ml pancreatic a-amylase, and **(E)** after 3 h enzymatic digestion test with 35 U/ml pancreatic a-amylase, 37°C, 75 rpm. Adapted from Dimantov et al. (10) with permission from Elsevier, Copyright 2004.

After the natural starch is treated with α-amylase and glucosidase, porous starch with good adsorption properties can be formed, and the probiotics can be loaded on the porous starch to improve the thermal performance and stability of the probiotics. The deep holes produced by the enzyme-modified natural starch increase its surface adhesion ability, allowing probiotics to enter the starch granules (56). The preparation of extruded starch microparticle containing resveratrol and α-amylase was studied to simplify the resveratrol encapsulation and give more lightstability to the active resveratrol (93). As shown in Figure 3, coatings from high amylose corn starch (HACS) were prepared on glass beads as a model for encapsulated core using fluidized bed technique. Addition of resistant starch (RS) was used to further improve the coatings stability to enzymatic digestion. Therefore, the potential use of HACS coatings as food grade enteric coatings for protection of core materials from dissolution in the stomach and release in the small intestine was demonstrated (10).

## Application of starch-based nano-microcapsules in functional foods

**Figure 4:**
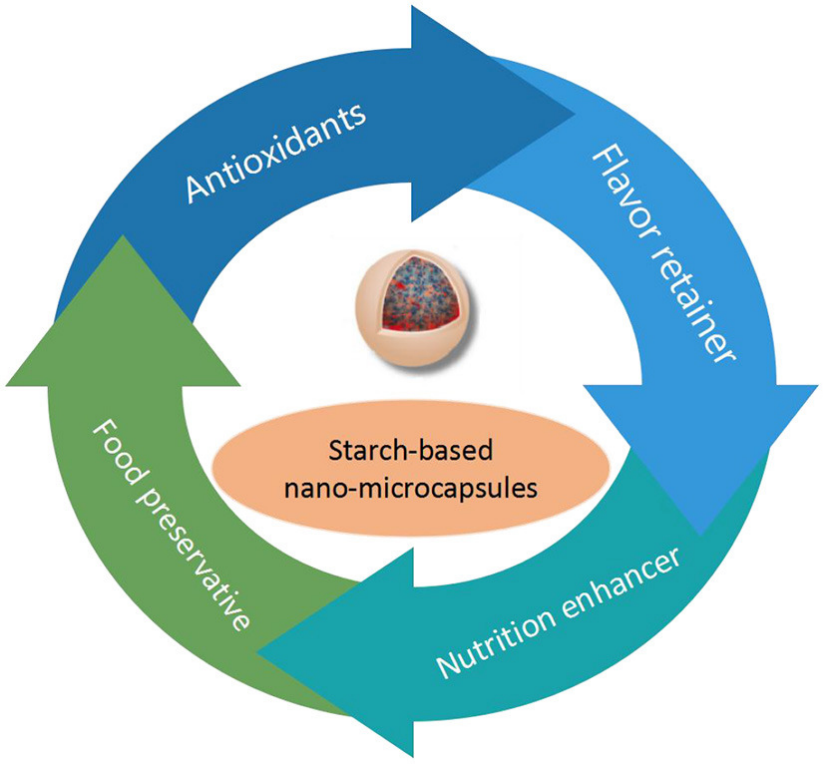
Application of starch-based nano-microcapsules in functional foods.

Starch-based nano-microcapsule technology has huge application potential in the field of functional foods. Different types of nano-microcapsules have different functional characteristics. As shown in Figure 4, according to the functional active ingredients embedded, the application of starch-based could be divided into four categories (antioxidants, flavor retainer, nutrition enhancer, and food preservative) (94, 95). Sources of wall and core materials and application of starch-based nano-microcapsules in functional foods were list in Table 3.

**Table 3:** Sources of wall and core materials and application of starch-based nano-microcapsules in functional foods.

**Wall materials**	**Core materials**	**Application in functional foods**	**References**
Highland barley starch	Cinnamon essential oil	Antioxidant and stabilizer of foodstuff	(33)
OSA-esterified taro starch	Avocado oil	Lipophilic bioactive compounds	(96)
Arrowroot starch compared with maltodextrin	Tuna fish oil	Oxidizable ingredient	(97)
Potato starch	Thyme oil	Chilled meat	(14)
Chayotextle (*Shechium edule Sw*.) starch	Ascorbic acid	Edible coatings of guava fruit	(98)
OSA-modified waxy corn starch and XG	Conjugated linoleic acid	Anti-diabetic, anti-adipogenic and anti-carcinogenic functions	(34)
Horse chestnut starch and β-cyclodextrin	Folic acid	Antioxidant	(26)
Resistant starch	Three probiotic strains (*Lactobacillus casei, Lactobacillus brevis and Lactobacillus plantarum*)	Oral Administration of probiotics	(99)

### Improving the stability of food antioxidants

Many antioxidative acitivity substances, such as phenols, flavonoids, anthocyanins, vitamins, and CLA, have great application potential in the functional food field, but they are easily affected by factors such as temperature and light, resulting in poor stability. Therefore, starch-based nano-microcapsule technology improves their stabilities and expands the scope of application (34, 63, 90). To evaluate enzymatically acylated rice starch as a wall material for encapsulation and release and to evaluate the effect on the antioxidant activity of phenolic compounds of gulupa seeds (Passiflora edulis. f. edulis), modified rice starch was used for compounds encapsulation. The capsules were characterized by their trapping efficiency, size, zeta potential, release parameters, and maintenance of the antioxidant activity *in vitro*. Trapping percentages >40 and 90% were observed by emulsion and aspersion, respectively (100). A starch with porous structure derived from purple sweet potato was prepared and used as a food-grade polymer for ultra-microencapsulation of olive oil. The results showed that compared with free olive oil, porous starch-based nano-microcapsules had a stable olive oil loading rate and significantly improved oxidation stability (78). Folic acid was easily degraded under acidic conditions, therefore, it should be protected during its passage through the gastrointestinal tract. The nano-microcapsules showed a higher transition temperature and melting temperature than free folic acid, which indicated that folic acid was thermally stable after encapsulation. The content of antioxidants and folic acid in intestinal juice was higher than that in gastric juice, ensuring a controlled release in the intestine (26). The effect of microencapsulated proanthocyanidins made by extruded starch (MPS) on the quality of chicken sausages stored at 4°C for 28 d was studied. The microstructure of the chicken sausage showed that the addition of MPS would not destroy the structure of the chicken sausage. Therefore, MPS was a potential natural antioxidant that could delay the oxidative deterioration and quality degradation of chicken sausages (101).

### Application in flavored food

In recent years, spices have played an important role in the development of functional foods. However, most of the spices contained in the spices are terpenoids, which were usually unstable in the presence of acid, light, oxygen or heat, and could undergo hydrolysis, rearrangement, polymerization and oxidation reactions to produce a series of harmful substances. The use of microencapsulation technology could embed some irritating or volatile and oxidizing substances in food to avoid direct stimulation of the human oral cavity. For substances with special fragrances, microencapsulation could extend their fragrance retention time and improve product quality (102). The combination of *S. edule* fruit starch (SS) with whey protein concentrate (WPC) and gum arabic (GA) was evaluate as a wall material for encapsulation of cinnamon oleoresin by spray drying (36). Embedding laughing flower extract in acetylated starch could effectively control the release of aroma in a simulated human saliva environment (102). Mustard flavor-starch nano-microcapsules were mixed with different concentrations of mustard flavor and core material, and evaluated their slow-release characteristics. The results showed that with the increase of humidity, the release rate of flavor increased, and the intensity of microencapsulated wasabi flavor in canned tuna was higher than that in the control group (103).

### Nutrient supplements in functional food

Most of the functional substances in food are absorbed in the small intestine, but before entering the small intestine, the strong acid and enzymes in the stomach limit the amount that can reach the small intestine (104, 105). The functionality of most substances is destroyed and it is difficult to enter the small intestine to exert its probiotics meta-function. Therefore, a challenging research in the food industry is to develop a delivery system for functional food ingredients, maintain the biological activity of the ingredients, and deliver them to the target digestive organs (91, 106). The cross-linking of starch can make it a potential wall material for targeted delivery of probiotics by changing its digestion effect. Three probiotic strains, namely *Lactobacillus casei, Lactobacillus brevis* and *Lactobacillus plantarum* were microencapsulated with resistant starch. The encapsulation efficiency (%) of resistant starch microspheres was between 43.01–48.46. The average diameter of resistant starch particles was in the range of 45.53–49.29 μm. The results showed that resistant starch was a potential carrier for oral administration of probiotics. The simulated release experiment *in vitro* proved that the starch-folic acid nano-microcapsules protected the folic acid during the passage through the gastrointestinal tract and improved the thermal stability of folic acid. Meanwhile, the free folic acid content in the intestinal juice was higher than that in the gastric juice, indicating that the nano-microcapsules had intestinal targeted release characteristics, so it could be added to foods for specific nutritional supplements for people or regions lacking folic acid (26).

### Application in food preservation

In the storage process of food, the growth of bacteria has a great impact on its shelf life and quality (14). Therefore, many foods use various sterilization methods, such as high temperature sterilization, ultraviolet sterilization lamps, etc., but the equipment is expensive and energy-efficient (107, 108). Some active substances with natural antibacterial effects such as curcumin, cinnamaldehyde, allicin, clove oil, etc. are more common in food additives, and have good effects on common pathogens such as staphylococcus aureus, vibrio cholerae, and salmonella enteritidis. However, these substances have shortcomings such as poor water solubility and low flavor threshold, which affect their antibacterial effect and flavor. The nano-microencapsulation technology includes these active substances, which can achieve the effect of food preservation (83, 87, 109–112). β-cyclodextrin assisted with porous starch was used as wall material, and its ratio of porous starch to β-cyclodextrin was 1/7. The allicin sample was added and dissolve in it to obtain its solution at concentration of 20%, which was then dripped into the above wall material solution and emulsified with sucrose ester. A nano-microencapsulation method of allicin was used to study the antiseptic effect on daily foods such as tofu, bread, chicken and pork. When the nano-microcapsules after the severe heat treatment were applied to the foods, their reduction rates of mold spores (Rm) increased only between 14.5 and 26.3%. The results showed that even after heat treatment, the nano-microcapsules still maintained a good sterilization effect and had potential application value in food antiseptic (111).

## Concluding remarks and future perspectives

The functional ingredients of food are embedded in starch-based nano-microcapsules. The premise is to maintain the stability of the core materials, including their resistance to external environments such as light, temperature, pH and strong stomach acid during storage. The starch-based nano-microcapsules deliver functional ingredients to the target location of the human body, such as the small intestine, colon, etc., to exert specific physiological effects. Various plant-derived starches can be modified by different methods to obtain wall materials with various characteristics, which can be used to embed functional ingredients in food (functional lipids, spices, probiotics, vitamins, etc.). Modifications of starch include oxidation, acetylation, hydrolysis, enzymes, etc. In particular, starch modified with hydrophobic groups can act as a surfactant, giving it a higher embedding rate. In addition, a variety of technologies can be applied to the preparation of food nano-microcapsules, such as spray drying, high-pressure homogenization, microwave, ultrasound, ultra-high pressure and so on. Research results showed that compared with traditional embedding agents such as gelatin, acacia gum, and xanthan gum, starch had many good properties that could be used to improve the quality of core materials, including improving antioxidant capacity, bioavailability, cell activity (probiotics), processing and storage stability, and concealing bad flavors.

At present, the research on the properties of nano-microcapsules is only limited to the characterization of their structural properties, and it is rarely used in actual food processing, and the practice in food production is slightly insufficient. The research on the structure of nano-microcapsules and the controlled release properties of core materials is limited, and they are limited to simply calculating the release amount in the simulated digestion model. There is a lack of systematic studies to illustrate the structure-activity relationship between the structure of nano-microcapsules and the controlled release properties. The *in vivo* digestion process and targeted sustained release mechanism of starch-based nano-microcapsules are still not very clear. This limits the control of their production process during the preparation of nano-microcapsules and their application in the food industry. Therefore, base on the key factors that affect the controlled release characteristics of the nano-microcapsules, the embedding rate and the stability of the nano-microcapsules need to be improved, and achieve their precise release in human organs and exert specific effects. The starch-based food nano-microcapsules can provide new ideas for the development of functional foods. In summary, the starch-based nano-microcapsule technology, as a new type of functional food technology, has an immeasurable market prospect.

## Author contributions

ST: conceptualization, funding acquisition, and writing-review and editing. XX: formal analysis, investigation, and writing-original draft. ZC: investigation and resources. XW: investigation, methodology, supervision, and writing-review and editing. All authors contributed to the article and approved the submitted version.

## Funding

The authors would like to acknowledge NSFC for financial assistance under NSFC Research Contract No. 31701636. This research was supported by the Cultivation Programme for Young Backbone Teachers in Henan Province (2021GGJS059) and funded by Natural Science Innovation Fund Support Program from Henan University of Technology (2021ZKCJ12). Special funded project for Natural Science Foundation of Henan (222300420423).

## Conflict of interest

The authors declare that the research was conducted in the absence of any commercial or financial relationships that could be construed as a potential conflict of interest.

## Publisher's note

All claims expressed in this article are solely those of the authors and do not necessarily represent those of their affiliated organizations, or those of the publisher, the editors and the reviewers. Any product that may be evaluated in this article, or claim that may be made by its manufacturer, is not guaranteed or endorsed by the publisher.
